# Clinicopathological analysis of thymic malignancies with a consistent retrospective database in a single institution: from Tokyo Metropolitan Cancer Center

**DOI:** 10.1186/1471-2407-14-349

**Published:** 2014-05-20

**Authors:** Yusuke Okuma, Yukio Hosomi, Kageaki Watanabe, Yuko Yamada, Hirotoshi Horio, Yoshiharu Maeda, Tatsuru Okamura, Tsunekazu Hishima

**Affiliations:** 1 Department of Thoracic Oncology and Respiratory Medicine, Tokyo Metropolitan Cancer and Infectious diseases Center Komagome Hospital, 3-18-22 Honkomagome, Bunkyo, Tokyo 113-8677, Japan; 2 Departments of Pathology, Tokyo Metropolitan Cancer and Infectious diseases Center Komagome Hospital, Bunkyo, Tokyo, Japan; 3 Departments of Thoracic Surgery, Tokyo Metropolitan Cancer and Infectious diseases Center Komagome Hospital, Bunkyo, Tokyo, Japan; 4 Department of Chemotherapy, Tokyo Metropolitan Cancer and Infectious diseases Center Komagome Hospital, Bunkyo, Tokyo, Japan; 5 Division of Oncology, Research Center for Medical Science, The Jikei University School of Medicine, Minato, Tokyo, Japan

**Keywords:** Thymoma, Thymic carcinoma, Thymic epithelial tumor, World Health Organization classification, Treatment, Prognostic factor, Rare cancer

## Abstract

**Background:**

Thymic epithelial tumors (TETs), which comprise thymoma and thymic carcinoma, are rare cancers with specific morphological and clinical features. Their clinical characteristics and outcomes have gradually been clarified by assessing large-scale, retrospective data obtained with international cooperation.

**Methods:**

The study is a retrospective review of 187 Japanese patients with TETs who attended our institution from 1976 to 2012. Relevant clinical features of patients with TETs and their tumors, including histology, staging, treatment strategies, and overall survival, were investigated. Differences in survival were assessed by the Kaplan–Meier method and uni- and multi-variate Cox proportional hazards regression analyses.

**Results:**

The 187 patients included 52 patients with stage I, 37 with stage II, 22 with stage III, and 76 with stage IVa/IVb tumors according to the Masaoka–Koga Staging System. As to histological type, five patients had type A, 33 type AB, 19 type B1, 39 type B2, and 15 type B3 thymomas, whereas 68 patients had thymic carcinoma, including 11 with neuroendocrine carcinomas according to the 2004 WHO classification. Either insufficient data were available to classify the tumors of the remaining eight patients or they had rare types. Immunological abnormalities were present in 26 patients, most of whom had thymomas (21.8% of the thymoma group). Most of the patients who presented with symptoms had myasthenia gravis or extensive thymic carcinoma. Secondary cancers were present in 25 patients (13.3%). The overall 5- and 10-year survival rates for thymoma were 85.4 and 71.5%, respectively, and those for thymic carcinoma were 33.8 and 2.3%, respectively. OS differed significantly between stage IVa thymomas and thymic carcinomas. The stage and whether the tumors were thymomas or thymic carcinomas were significant determinants of survival according to multivariate analysis.

**Conclusion:**

The efficacy of treatments for thymoma and thymic carcinoma should be investigated separately because these tumors differ in their clinical features and prognosis.

## Background

Thymic epithelial tumors (TETs, or thymic malignancies) which comprise thymoma and thymic carcinoma, are rare cancers according to the definition of the RARECARE project, which is supported by the European Commission. Their annual incidence is approximately 0.15 cases in the United States [1] and 0.32 cases in the Netherlands [2] per 100,000 person-years. Thymic malignancies are extremely heterogeneous, with an exceedingly broad spectrum of morphological appearances and immunological abnormalities. Because thymomas are bioactive and have organotypic features that lead to autoimmune manifestations, whereas thymic carcinomas are not immunologically active and lack organotypic features, patients with thymic carcinoma usually have symptoms associated with tumor extension or metastasis.

Because of their rarity, the clinical characteristics and prognostic indicators in patients with thymic malignancies have not been well characterized [3]. Therefore, the International Thymic Malignancy Interest Group (ITMIG) was organized. Despite the paucity of evidence, this group has reached consensus agreements in support of some treatment modalities, having conducted some single-arm phase II studies and a few retrospective studies of small groups of treated patients with diverse backgrounds [4]. However, the optimal therapeutic strategy remains controversial. In previous studies, patients with thymoma and thymic carcinoma have basically received the same treatment. However, it has recently been suggested that the two types of tumors should be considered separate entities [5]. In addition, the ITMIG has proposed using the Masaoka–Koga staging system [6] and the 2004 World Health Organization (WHO) histological classification; both proposals have been accepted [7]. Thus, we believe it is necessary to review and clarify the nature and characteristics of these clinical entities in light of the proposed criteria. Furthermore, the National Comprehensive Cancer Network has updated its guidelines for the clinical management and treatment of thymic malignancies, despite their rarity [8].

The objective of the present study was to retrospectively clarify the clinical characteristics, prognosis, and prognostic indicators of patients with thymoma and thymic carcinoma according to the 2004 WHO classification [7] who had attended our institution over a 30-year period.

## Methods

### Database

This is a retrospective review of patients diagnosed with thymic malignancies between January 1976 and December 2012 identified from the databases at Tokyo Metropolitan Cancer and Infectious diseases Center Komagome Hospital (Tokyo, Japan). The codes of the International Classification of Diseases (9th edition) were used.

This retrospective study was approved by the Ethics Committee of the Tokyo Metropolitan Cancer and Infectious diseases Center Komagome Hospital (#1049).

### Patients and histological evaluation

A retrospective review of relevant clinical features and treatment-related data of 187 consecutive Japanese patients with diagnoses of thymic malignancies was performed. Their pathology was reviewed by a thoracic pathologist (TH) according to the 2004 WHO classification and Masaoka–Koga staging system [6]. Diagnoses of thymic carcinoma were confirmed by hematoxylin-eosin staining and immunohistochemistry for CD5 and/or CD117 (c-KIT) and/or p63 to exclude other malignant thoracic tumors, as well as supplemental testing for terminal deoxynucleotidyl transferase to distinguish carcinomas from thymomas. Clinical factors were also examined. Data were collected in accordance with the Standard Definitions and Policies of the ITMIG [4].

Clinical factors including age, sex, histological subtype, stage, immunological abnormalities, secondary malignancies, initial treatment -intent of modality, and survival were examined, relevant data having been obtained from medical records and laboratory data. Staging had been determined according to the Masaoka–Koga staging system by computed tomography, magnetic resonance imaging, positron emission tomography, or bone scanning. Histology was also classified according to the 2004 WHO classification. The patients had been treated with curative-intent or palliative-intent surgery, radiotherapy, chemotherapy, and best supportive care, or a combination of these modalities.

### Statistical analysis

Descriptive statistics were used to summarize the patients’ baseline characteristics. Survival time was defined as the period from the date of initiation of initial treatment (surgery, radiotherapy, chemotherapy, or best supportive care) to the date of death from any cause or last follow-up. The Kaplan–Meier method was used to assess overall survival and 5- and 10-year survival rates. Patients who had been lost to follow-up were censored at the time of last contact. These end points reflected clinical practice because of the retrospective nature of the data. In accordance with the ITMIG Standard Definitions and Policies, the 5-year survival rate of patients with thymic carcinomas and 10-year survival rate of those with thymoma were calculated. Correlations between histological subtype according to the 2004 WHO classification and Masaoka–Koga stage were evaluated using a nonparametric measure of statistical dependence between the two variables.

The log-rank test was used to identify prognostic indicators by uni- and multi-variate analysis. Candidate variables analyzed included age (<70 vs. ≥70 years), sex (male vs. female), staging, immunological abnormalities, secondary malignancies, and histological subtype according to the WHO classification 2004. Significance according to univariate analysis and multivariate Cox proportional hazard models was defined as *p* < 0.05. All statistical analyses were performed using JMP9 (SAS Institute, Cary, NC, USA).

## Results

### Characteristics of patients with thymoma and thymic carcinoma and their tumors

Of the 187 patients, 119 (52 men, 67 women) had thymomas and 68 (38 men, 30 women) had thymic carcinomas. Their median age was 58 years for thymoma and 63 years for thymic carcinoma. As to histology, according to the 2004 WHO classification five patients had type A, 19 type B1, 39 type B2, 15 type B3, and 33 type AB thymomas. Of the 68 patients with thymic carcinoma, 11 (16.2%) had neuroendocrine carcinomas (three small cell carcinomas, two large cell neuroendocrine carcinomas, and six carcinoid tumors), 46 squamous cell carcinomas (67.6%), five mucoepidermoid carcinomas (7.4%), and one a lymphoepithelioma-like carcinoma. The remaining eight patients either had other histological types or relevant data were unavailable. Only a patient with thymic carcinoma had autoimmune-related manifestations. Most of the patients who presented with symptoms had myasthenia gravis or thymic carcinoma. Secondary malignancies were seen in 25 patients (13.4%). At the time of diagnosis, 52 thymoma patients (43.7%) had stage I, 31 (26.1%) stage II, 12 (10.1%) stage III, and 24 (20.1%) stage IVa/IVb according to the Masaoka–Koga Staging System, whereas six thymic carcinoma patients (8.8%) had stage II disease, 10 (14.7%) stage III disease, 16 (23.5%) stage IVa disease, and 36 stage IVb disease (52.9%). A variety of immunological paraneoplastic abnormalities were observed in the 26 patients with thymomas (21.8%). There was some overlap among patients with immunological abnormalities.

Relevant patients’ characteristics are summarized in Table 1. The median follow-up for all 187 patients at the time of analysis was 43.9 months (range: 0.3–404.8 months).

**Table 1:** Characteristics of patients and tumors in patients with thymic malignancies

**Characteristics**		**Thymoma**		**Thymic carcinoma**	
		***n*** **= 119**	**(%)**	***n*** **= 68**	**(%)**
Median age,	Years [range]	58 [26–81]	-	63 [14–83]	-
Gender					
	Male	52	43.7	38	55.9
	Female	67	56.3	30	44.1
Histology					
	*Thymomas*	119	100.0		
	Type A	5	4.2		
	Type B1	19	16.1		
	Type B2	39	32.8		
	Type B3	15	12.6		
	Type AB	33	27.7		
	Other subtypes or missing data of thymoma	8	6.7		
	*Thymic carcinomas*			68	100.0
	Squamous cell carcinoma			46	67.6
	Mucoepidermoid carcinoma			5	7.4
	Lymphoepithelioma-like carcinoma			1	1.5
	Undifferentiated carcinoma			3	4.4
	Neuroendocrine carcinomas			11	16.2
	*Small cell carcinoma*			*3*	*4.4*
	*LCNEC*			*2*	*2.9*
	*Carcinoid*			*6*	*8.8*
	Not classified in WHO classification			2	2.9
Staging					
	I	52	43.7	0	0
	II	31	26.1	6	8.8
	III	12	10.1	10	14.7
	IVa	18	15.1	16	23.5
	IVb	6	5.0	36	52.9
Complications					
	*Immunological abnormalities (overlapped)*				
	Myasthenia gravis	20	16.8	1	1.5
	Pure red cell aplasia	4	3.3		
	Hypogammaglobulinemia	5	4.2		
	*Secondary malignancies*	12	10.1	13	19.1
Initial treatment-intent of modalities					
	*Curative-intent treatment*	116	97.5	43	63.2
	Surgery	109	91.6	30	44.1
	Surgery alone	89	74.8	10	14.7
	Surgery with perioperative treatment	20	16.8	20	29.4
	Radiotherapy	7	5.9	13	19.1
	Definitive radiotherapy alone	1	0.8	1	1.5
	Chmoradiotherapy (sequential/concurrent)	6	5.1	12	17.6
	*Palliative-intent treatment*	3	2.5	25	36.8
	Chemotherapy alone	2	1.7	24	35.3
	Best supportive care	1	0.8	1	1.5

### Treatment modalities and strategies for thymoma and thymic carcinoma

Initial treatment was performed with curative-intent in 97.5% of patients with thymoma (surgery in 91.6%, radiotherapy in 5.9%) and in 63.2% of those with thymic carcinoma (surgery in 44.1%, radiotherapy in 19.1%).

The types of treatment modality are also summarized in Table 1.

### Clinical outcomes of thymoma and thymic carcinoma by stage and histological classification

#### Stage

**Figure 1:**
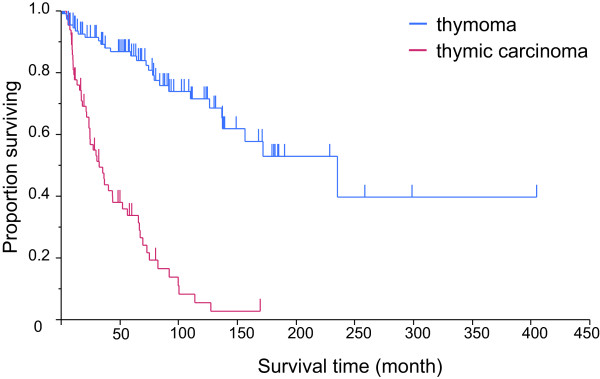
**Kaplan–Meier curves showing median overall survival for thymoma (n = 119) was 235.2 months (95% CI, 137.3-not reached) and for thymic carcinoma (n = 68) 32.4 months (95% CI, 23.7–52.2) (*p*** **< 0.0001).** The 5 year-survival for thymoma and thymic carcinoma was 85.4 and 33.8%, respectively.

**Figure 2:**
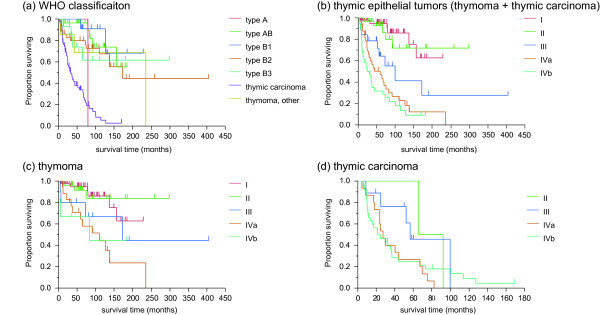
**Survival curves by histological subtypes. (a)** Kaplan–Meier survival curves for each histological subtype of the WHO classification. **(b)** Overall survival by stage for patients with thymic malignancies. **(c)**, **(d)** stratified by stage in thymoma and thymic carcinoma

The overall median OS of patients with thymoma was 235.2 months (95% CI, 137.3-not reached), whereas that of those with thymic carcinoma was 32.4 months (95% CI, 23.7–52.2) (*p* < 0.0001) (Figure 1). The survival of patients with stages I, II, III, IVa, and IVb thymoma was not reached, not reached, 171.8, 110.1, and 83.8 months, respectively. The 5- and 10-year survival rates of patients with thymoma were 85.4 and 71.5%, respectively (Figure 2b-d). Conversely, survival of patients with stages II, III, IVa, and IVb thymic carcinoma was 78.9, 56.4, 27.3, and 21.7 months, respectively (Figure 2b-d). The 5- and 10-year survival rates of patients with thymic carcinoma were 33.8 and 2.3%, respectively.

#### Histological classification

The 5- and 10-year survival rates were 100% and censored for patients with type A, 96.3 and 73.8% for those with type AB, 90.9% and 68.2 for those with type B1, 79.8 and 67.3% for those with type B2, and 61.6 and 61.6% for those with type B3, respectively (Figure 2a). The median OS of patients with thymic carcinoma was 36.4 months (95% CI, 23.7-52.2) for all stages combined, whereas the 5- and 10-year survival rates were 36.3 and 2.3%, respectively. The median OS of patients with high-grade histology was 24.7 months, whereas that for patients with low-grade histology was 36.8 months. The overall median survival of patients with neuroendocrine carcinoma was 43.9 months. The median survival of the six patients with well-differentiated neuroendocrine carcinoma was 36.4 months (95% CI 7.5–92.0) with a 5-year survival of 20.0%, whereas it was 43.9 months (95% CI 5.6–127.4) in the five patients with poorly-differentiated neuroendocrine carcinoma, with a 5-year survival of 20.0%.

### Correlation between tumor type according to the 2004 WHO classification and Masaoka–Koga stage

The distribution of the WHO classification and Masaoka–Koga stage of the 187 patients is shown in Table 2. The proportions of advanced stages (Masaoka–Koga stages III, IVa, and IVb) increased gradually from Type A thymoma to thymic carcinoma. There was a significant correlation between the WHO classification and Masaoka–Koga stage (Spearman’s rank correlation coefficient = 0.69, *p* < 0.0001).

**Table 2:** Relationships between overall survival and WHO histological subtype according to the Masaoka–Koga staging system

**WHO classification (2004)**	**N**^**o **^**of Pts**		**Masaoka-Koga Stages**					**Survival rates (%)**	
		**(%)**	**I**	**II**	**III**	**IVa**	**IVb**	**5-yr OS**	**10-yr OS**
Thymoma	119		52	31	12	18	6	85.4	71.5
A	5	2.7	4	1	0	0	0	100	-
AB	33	17.6	21	8	1	2	1	96.3	73.8
B1	19	10.2	11	5	0	2	1	90.9	68.2
B2	39	20.9	12	9	8	8	2	79.8	67.3
B3	15	8.0	1	5	3	5	1	61.6	61.6
Thymoma, other	8	4.3	3	3	0	1	1	68.6	68.8
Thymic carcinoma	68	36.4	0	6	10	16	36	33.8	2.3
Thymic carcinoma excluding NEC	57	30.5	0	5	9	13	30	33.0	4.1
NETT	11	5.9	0	1	1	3	6	27.3	9.1
Total	187	100.0	52	37	22	34	42	65.9	45.3

WHO, World Health Organization; OS, overall survival; NETT, neuroendocrine thymic tumors.

### Prognostic factors affecting survival according to uni- and multi-variate analysis

According to univariate analysis, age and all Masaoka–Koga stages were significantly correlated with survival in patients with thymoma. However, this was not the case in those with thymic carcinoma. According to multivariate analysis, early stages (Masaoka-Koga stage I or II) and advanced stages (IVa or IVb) of both thymomas and thymic carcinomas correlated significantly with survival (Table 3).

**Table 3:** Uni- and multi-variate analysis of survival in patients with thymic malignancies

**Univariate analysis**						
**Variants**	***Thymoma***			***Thymic carcinoma***		
	**n**	**MST [95% CI]**	***p*-value**	**n**	**MST [95% CI]**	***p*-value**
Age (y)			0.0018*			0.36
<70	91	71.3 [54.4-88.1]		50	24.1 [13.5-43.6]	
≥70	28	30.1 [13.8-59.9]		18	24.6 [11.9-36.4]	
Gender			0.29			0.076
Male	52	235.2 [136.8-NR]		38	29.7 [13.5-40.2]	
Female	67	171.8 [110.1- NR]		30	56.4 [23.7-73.0]	
Masaoka-Koga Stage			0.0012*			0.18
I	52	NR [136.8- NR]		0	-	
II	31	NR [NR - NR]		6	78.9 [65.8-92.0]	
III	12	171.8 [4.7- NR]		10	56.4 [8.8-99.6]	
IVa	18	110.1 [33.8-235.2]		16	27.3 [17.2-43.9]	
IVb	6	83.8 [0.6- NR]		36	21.6 [11.3-36.4]	
Immunological abnormalities			0.26			0.59
Yes	26	NR [171.8- NR]		2	71.2 [69.5-73.0]	
No	93	156.6 [126.3- NR]		66	30.4 [23.6-43.9]	
Secondary Malignancies			0.42			0.54
Yes	12	235.2 [74.4-235.2]		13	36.8 [5.8-65.8]	
No	107	171.8 [136.8- NR]		55	30.4 [21.6-56.4]	
WHO classification [thymoma]			0.68			
A	5	79.2 [-]				
AB	33	- [91.6- NR]				
B1	19	- [126.3- NR]				
B2	39	171.8 [110.1- NR]				
B3	15	- [37.2- NR]				
Other	8	235.2 [4.4-235.2]				
[thymic carcinoma]						0.95
High grade				52	36.8 [23.7-65.8]	
Low grade				16	24.7 [8.5-66.6]	
Multivariate analysis						
Variants	*Thymoma*			*Thymic carcinoma*		
	HR	95% CI	*P*-value	HR	95% CI	*p*-value
Staging	IVa/ I: 4.62	[1.78-13.37]	0.016*	II/IVa: 0.27	[0.43-0.99]	0.048*
	IVa/II: 6.40	[2.03-28.17]	0.001*			
	IVb/II: 5.56	[1.02-30.20]	0.047*			
Immunological abnormalities	2.0	[0.73-6.35]	0.18			
Secondary Malignancies	4.4	[0.63-51.9]	0.26	1.36	[0.57-3.28]	0.44
WHO Classification						
(A/B1/B2/B3/AB/other)	none	none	none	-	-	-
(high grade vs. low grade)	-	-	-	0.88	[0.46-1.57]	0.70
(thymic ca. vs. thymoma)	6.7	4.1-11.1	< .0001*	-	-	-

NR, not reached; CI, confidence interval; MST, median survival time; HR, hazard ratio; CI confidence interval; *p < 0.05.

## Discussion and conclusion

The present retrospective analysis examined the clinical outcomes of 187 patients with thymic malignancies. The clinical characteristics and outcomes in these unselected subjects were similar to those previously reported from large, multi-institutional series.

Based on the Müller–Hermelink classification [9], the WHO classification of thymomas was first proposed in 1999 [10]. In the 2004 WHO classification, thymic carcinoma, including neuroendocrine carcinoma, was separated from thymoma and given a new category [7]. Thymomas are classified into five groups: A, AB, B1, B2, and B3. According to retrospective studies, Types A and AB have a better prognosis than B1, B2, B3, and carcinomas [11-13]. In contrast, other studies have failed to identify a correlation between survival and WHO classification [14-16]. These results were discussed as limitations owing to the difficulty in accurate reproducibility when diagnosing thymic malignancies with the WHO schema. However, clinical features such as immunological abnormalities and secondary malignancies may contribute to prognosis. Although our results did not demonstrate a significant association between abnormalities or secondary malignancies and survival, some studies have reported that types A and AB thymoma have a low association with myasthenia gravis, whereas types B1 and B2 are more likely to be associated with myasthenia gravis. Up to 45% of patients with thymoma develop myasthenia gravis [17,18]. According to the WHO classification, there are 13 subtypes of thymic carcinoma; 60–70% of all thymic carcinomas being subtypes of squamous cell carcinoma and lymphoepithelioma-like carcinoma. Recent biomarker investigations have explored c-KIT as a characteristic of thymic carcinoma [19]. Clinically, thymomas and thymic carcinomas have different patterns of recurrence: thymomas mainly result in pleural dissemination as opposed to the distant metastases characteristic of thymic carcinoma [20]. The WHO classification still has some limitations, in that distinguishing even thymoma and thymic carcinoma subtypes remains difficult. As to staging systems, the Masaoka–Koga staging system is widely accepted for both thymoma and thymic carcinoma, which is problematic because incorrect diagnoses, confounding of clinical entities, and intermingled management tend to occur. Till today, only a few studies according to the 2004 WHO Classification and Masaoka-Koga stage have been published (Table 4).

**Table 4:** Previously reported and present study survival rates of patients with thymic malignancies by Masaoka–Koga stage and 2004 WHO Classification

**Reference**	**No. of pts**	**MST (mo)**	**5-yrs survival in each stage (%), (frequency, %)**					**5-yrs survival (%)**	**10-yrs survival (%)**	**Prognostic factor**
			**I**	**II**	**III**	**IVa**	**IVb**			
de Jong et al. [2]	203*	N/A	82.9 (12.3)	87.8 (36.9)	57.6 (26.1)	55.6 (24.6)		69	40	Resection, Age
										WHO classification
										Masaoka disease stage
Masaoka et al. [29]	93*	N/A	92.6 (39.8)	85.7 (13.8)	69.6 (34.4)	50.0 (11.8)		74.1	57.1	N/A
Mariano et al. [30]	171*	N/A	93.3 (9.4)	88.7 (44.4)	74.6 (27.5)	43.4 (18.7)		N/A	N/A	Masaoka-Koga stage
										thymoma vs. thymic ca
JART study [31]	2807	N/A	97.7 (34.6)	95.8 (36.0)	85.8 (15.7)	72.8 (6.5)	57.4 (5.2)	N/A	N/A	N/A
Present study	187	99.6	NR (27.3)	NR (20.3)	99.6 (12.1)	59.2 (18.2)	24.8 (22.5)	65.9	45.3	Masaoka-Koga Stage II
										thymoma vs. thymic ca

pts, patients; MST, median survival time; yrs, years; N/A, not available; *available data on survival is summarized.

The present study is based on a relatively large database including all treated cases from the department of surgery, medical oncology, and radiation oncology. Additionally, we found that thymomas and thymic carcinomas exhibited a variety of clinical behaviors as reported in the past study. We believe that our single-institution data are reliable in that all cases were diagnosed by a pathologist who authored the thymic carcinoma section of the WHO classification book [7]. The lack of correlation between survival and WHO classification in thymomas may be attributable to the small numbers of patients studied, immunological abnormalities, or too few events because of the characteristically long survival. In our study, patients with thymomas had a similar prognosis to that previously reported, whereas both indolent and aggressive clinical courses occurred in patients with thymic carcinomas, including thymic squamous cell carcinoma. In neuroendocrine thymic tumors (or carcinomas) (NETT), the present small cohort of well-differentiated and poorly differentiated NETT showed similar clinical behavior to that reported in previous studies [21]. In the present study, no patients with NETT developed multiple endocrine neoplastic syndrome. As previously reported for NETT, the prognosis in this subgroup was poor [22]. The clinical entity of NETT is gradually becoming better known: the European Society of Medical Oncology has already published guidelines for NETT [23].

The key limitation of the present study was the small numbers of patients in each stage of thymoma or thymic carcinoma, resulting in a paucity of data compared with that obtained in randomized trials. However, this is a common limitation of studies of rare cancers. Second, we were unable to follow patients up, particularly young patients with thymoma or early stages of carcinoma whose tumors had been resected and who had no immunological abnormalities. Thus, there were more censored patients in the thymoma cohort than in the carcinoma cohort.

Large-scale databases are being established in Japan, the USA, and Europe as a first step toward conquering thymic malignancies. This approach appears to be a role model for studying rare diseases. Because these databases are drawn from surgical cases, they will provide little data on the clinical entities of thymic malignancies. Therefore, single-institution databases, such as that used in this study, are still meaningful because of the consistency of treatment and pathological evaluation; the latter would result in more reliable and reproducible diagnoses of thymic malignancies. Nevertheless unified, multi-institutional databases centered on the ITMIG are indispensable. Studies using such databases will clarify the clinical entities of and evolve treatment strategies for rare cancers such as thymic malignancies, which tend to fall behind in treatment development compared with common cancers. To minimize the biases from limited data concerning the reliability of diagnosis or treatment, every strategy must be carried out to overcome obstacles owing to the rarity of the cancer.

Plans are being made for prospective clinical trials on this rare cancer. However, the inevitably small sample size of future phase II studies will likely mean they have insufficient power to establish that findings are significant. In addition, as Weksler et al. have pointed out, a fundamental problem still remains in that the diagnosis of thymic malignancies, especially thymic carcinomas, is difficult [24]. In fact, in the WJTOG 4207L trial [25], 25% of patients diagnosed with thymic carcinoma by local hospitals were found to have incorrect diagnoses when centrally reviewed. Thus, central review of diagnoses is essential and the results of such studies must be interpreted with care. Investigators who plan clinical trials of thymic malignancies should incorporate central review by reliable pathologists who have experience with thymic malignancies. The importance of central review in clinical trials on rare cancers was demonstrated in the multi-institutional clinical trial of imatinib for c-Kit or platelet-derived growth factor receptor (PDGFR) positive sarcoma. In this trial, the concordance rate between the trial sites and central review for immunohistochemical staining was only 63.3% [26]. Also, the guidelines for gastrointestinal stromal tumors (GISTs) recommend taking care with the diagnosis of c-Kit-negative GIST, which requires consulting a specialist in GISTs who has experience in additional antibody staining or *c-Kit* or *PDGFR* gene analysis [27,28].

In summary, the further clinical management of thymoma and thymic carcinoma should be investigated separately because of the clinical differences between thymoma and thymic carcinoma. Moreover, a detailed population-based series that highlights the many challenges clinicians face when treating thymic malignancies, for which little evidence-based data concerning therapy is available. Also, the advantages and disadvantages of a single-institutional database, especially on rare cancers, such as that used in this study, have been discussed. Although there have been advances in surgical techniques, radiation planning, systemic therapy, and supportive care for patients with thymic malignancies, more research and collaborative efforts are needed to produce evidence-based guidelines. International database projects and multidisciplinary meetings supported by the ITMIG will undoubtedly help fulfill this need.

### Ethics statement

This study was approved by the Ethics Committee of Tokyo Metropolitan Cancer and Infectious diseases Center Komagome Hospital (Tokyo, Japan), and conducted in accordance with the Declaration of Helsinki.

## Competing interests

The authors declared that they have no competing interest.

## Authors’ contributions

YO, YH, and KW collected data and established a database for thymic malignancies. YO drafted the manuscript. HH, YM, and TO provided surgeons’ and medical oncologists’ perspectives. YY and TH examined specimens of thymic malignancies and provided opinions from a pathology perspective. YH conceived of the study, participated in its design and coordination, and helped to draft the manuscript. All authors have read and approved the final manuscript.

## Pre-publication history

The pre-publication history for this paper can be accessed here:

http://www.biomedcentral.com/1471-2407/14/349/prepub
